# Proteomic Analysis of the Effect of Fuzheng Huayu Recipe on Fibrotic Liver in Rats

**DOI:** 10.1155/2013/972863

**Published:** 2013-01-29

**Authors:** Hongdong Xie, Yanyan Tao, Jing Lv, Ping Liu, Chenghai Liu

**Affiliations:** ^1^Institute of Liver Diseases, Shuguang Hospital, Shanghai University of Traditional Chinese Medicine, Shanghai 201203, China; ^2^Taizhou Municipal Hospital, Taizhou, Zhejiang 318000, China; ^3^E-Institute of TCM Internal Medicine, Shanghai Municipal Education Commission, Shanghai 201203, China; ^4^Shanghai Key Laboratory of Traditional Chinese Clinical Medicine, Shanghai 201203, China

## Abstract

Hepatic fibrosis is a common pathological process of chronic liver diseases and would lead to cirrhosis, and Fuzheng Huayu (FZHY) is an effective Chinese herbal product against liver fibrosis. This study observes FZHY influence on proteome of fibrotic liver with differential proteomic approach and aims to understand FZHY multiple action mechanisms on liver fibrosis. The liver fibrosis models were induced with intraperitoneal injection of dimethylnitrosamine for 4 weeks in rats and divided into model control (model) and FZHY-treated (FZHY) groups, while normal rats were used as normal control (normal). After model establishment, rats in FZHY groups were administered 4 g/kg wt of FZHY for 4 weeks, and normal and model groups were given the same volume of saline. The liver proteins in the above 3 groups were separated by two-dimensional gel electrophoresis (2-DE), the differentially expressed spots were analyzed and compared between normal and model or model and FZHY groups, and then the proteins were identified with mass spectrum analysis and validated partially with western blot and real-time PCR. 1000*~*1200 spots were displayed on each 2D gel, and a total of 61 protein spots were found with significant intensity difference between normal control or FZHY and model control. 23 most obviously differential spots were excised, and in-gel digestion and 21 peptide mass fingerprints (PMF) were obtained with MALDI-TOF MS analysis, and 14 proteins were identified through protein database searching. Among 14 differentially expressed proteins, 8 proteins in normal and FZHY groups had the same tendency of differential expression compared with the ones in model group. And one of them, vimentin, was validated by western blot and real-time PCR analyses. Our study reveals 12 proteins responsible for fibrogenesis induced by DMN in rats, and among them, 8 proteins in fibrotic liver were regulated by FZHY, including aldehyde dehydrogenase, vimentin isoform (CRA_b), gamma-actin, vimentin, fructose-bisphosphate aldolase B, aldo-keto reductase, S-adenosylhomocysteine hydrolase isoform, and HSP90. It indicates that the action mechanism of FZHY antiliver fibrosis may be associated with modulation of proteins associated with metabolism and stress response, as well as myofibroblast activation. The study provides new insights and data for exploring the liver fibrogenesis pathophysiology and FZHY action mechanism against liver fibrosis.

## 1. Introduction

Liver fibrosis and cirrhosis represent the consequences of a sustained wound healing response to chronic liver injury from a variety of causes, including viral, autoimmune, drug-induced, cholestatic, alcoholic, and metabolic diseases [1]. The matrix component of the scar tissue in cirrhosis is similar regardless of its etiologies [2]. These scar constituents accumulate from a net increase in liver extracellular matrix (ECM) components, regulated mainly by hepatic stellate cells (HSCs), which are mediated by various cytokines, growth factors, and proteases and their inhibitors [3, 4]. Recent decades had witnessed the significant progress in the understanding of liver injury and fibrosis; however the efficient, and well-tolerated antifibrotic drugs are still missing, the mechanisms of liver fibrogenesis is not fully elucidated and specific anti-fibrotic drug targets are still lacking [5]. Therefore, it is very important to further explore the molecular mechanism of liver fibrosis, in particular, to seek effective and safe medicines for liver fibrosis treatment.

There is a recent increasing interest in Traditional Chinese medicine (TCM) or other botanic medicines, which have been used for thousands of years because of their clinic efficacy and easy applicability, in particular in the field of liver diseases. However, TCM usually consists of complex mixtures and compositions of herbs and apparently exerts its action through multiple pathways [6]. It is difficult to understand the complicated action mechanisms of TCMs fully with conventional methodologies such as western blot analysis, which can semiquantitatively determine the expression of proteins of interest [7]. Proteomics and other system biology approaches could simultaneously generate large biological data sets and provide powerful tools for the understanding of the mechanisms of TCM [8]. Especially, the comparison of the expressional proteome between normal and diseased sample (cells or tissue) or between the diseased and treated sample would be very helpful to explore disease- or drug- specific differential proteome and can lead to identify the molecular targets involved in different pathophysiological states of the diseases and to understand the complex action mechanisms of medicines including TCM. 

Fuzheng Huayu recipe (FZHY), a prescription usually used for treating liver fibrosis in traditional Chinese medicine (TCM), is made of six traditional Chinese drugs: *Radix Salvia Miltiorrhizae* (Danshen), *Cordyceps* (Chongcao), *Semen Persicae, Gynostemma Pentaphyllammak* (Jiaogulan), *Pollen Pini* (Song hua fen), and *Fructus Schisandrae Chinensis* (Wuweizi) [9]. Our previous studies suggested that the recipe could significantly alleviate liver fibrosis in animal models through anti-inflammation, antioxidative stress, antiproliferation, and activation of hepatic stellate cells (HSCs), protection of liver function, decreasing the collagen synthesis and promoting degradation of extracellular matrices (ECM) [10–15]. Additionally, a multicenter, randomized, double-blinded, and parallel control experiment demonstrated that FZHY had good therapeutic effects on improving liver fibrosis due to chronic hepatitis B [16]. The six herbs containing recipe are complex mixtures of ingredients, which act in concert to treat imbalanced body symptoms, likely with the mechanisms of simultaneously treating multiple therapeutic targets [9]. Albeit a great deal has been done to understand the therapeutic mechanism of the detailed mechanism is still unclear. In the present study we used contemporary proteomics tools to compare the differences in protein patterns of liver from normal, DMN-induced fibrotic rats and FZHY-treated rats. Furthermore, the action mechanism of FZHY antifibrosis of liver is discussed.

## 2. Materials and Methods

### 2.1. Chemicals

Dithiothreitol (DTT), urea, agarose, glycerol, bromophenol blue, 3-[(3-cholamidopropyl)-dimethylammonio]-1-propanesulfonate (CHAPS), mineral oil, acrylamide, bisacrylamide (Bis), trisbase, glycine, sodium dodecyl sulfate (SDS), ammonium persulphate, and N,N,N,N- tetramethylene diamine (TEMED) were from Bio-Rad (Hercules, CA, USA). Immobiline Dry Strip gels (pH 3–10 nonlinear) and IPG Buffer solutions (pH 3–10 nonlinear) were from Amersham Biosciences (Uppsala, Sweden). Iodoacetamide (IAA), ammonium bicarbonate, formic acid, and *α*-Cyano-4-hydroxycinnamic acid (CHCA) were from Sigma (St. Louis, MO, USA). Acetonitrile (ACN) and methanol were from Fisher Scientific (Fair Lawn, New Jersey, USA). Trifluoroacetic acid (TFA) was from Merck (Schuchardt, Hohenbrunn, Germany). Trypsin (sequencing grade) was purchased from Promega (Madison, WI, USA). All buffers were prepared with Milli-Q water (Millipore, Bedford, MA, USA).

### 2.2. Materials

FZHY was prepared by Shanghai Sundise Medicine Technology Development Co. Ltd., China, (SFDA approval no.: z20050564). The recipe consists of six crude herbs with one-day dose for the adults: 8.0 g of *Radix Salviae Miltiorrhizae*, 4.0 g of *Fermentation Mycelium Powder*, 2.0 g of *Fructus Schisandrae Chinensis*, 2.0 g of *Semen Persicae*, 2.0 g of *Pollen Pini*, and 6.0 g of *Gynostemma Pentaphyllammak *(Table 1). The FZHY powder was made, and major ingredients were determined by Shanghai Sundise Medicine Technology Development Co. Ltd. for quality control (Table 2). In the present study, FZHY powder was suspended in distilled water at a concentration of 0.5 g/mL for administration to animals. FZHY was administrated daily by intragastric gavage at a dose of 4.0 g (crude drug)/kg body weight.

### 2.3. Animals

Male SD rats of weighting 120–150 g were used. The rats were fed with standard rat diet and water according to the guidelines approved by the Chinese Association of Laboratory Animal Care. The liver fibrosis models were induced with intraperitoneal injection of dimethylnitrosamine (DMN, Tokyo Kasei Kogyo Co., Ltd., Tokyo, Japan) at a dosage of 10 *μ*g/kg body wt for consecutive 3 days weekly and totally for 4 weeks [17]. After DMN intoxication, model rats were divided into model control (model, *n* = 10) and FZHY treated (FZHY, *n* = 10) groups, while normal rats were used as normal control (normal, *n* = 8). After model establishment, FZHY groups orally took 4 g/kg wt of FZHY for 4 weeks, and the normal and model control groups took the same volume of saline. At the end of the treatment, all rats were sacrificed and their blood and liver tissues were collected. A portion of liver tissues was fixed in 10% phosphate-buffered formalin for histological studies after paraffin embedding. The remainder was snap-frozen in liquid nitrogen and stored at −80°C for Hyp content determination and protein extractions.

### 2.4. Measurement of Serum ALT and Albumin

Activity of ALT and albumin level were determined following the manufactures' instructions.

### 2.5. Measurement of Hepatic Fibrosis

The liver sections fixed in 10% phosphate-buffered formalin were embedded in paraffin, sectioned, and then stained with sirus red for collagen distribution. The content of hydroxyproline was determined by using Jamall's methods. Briefly, hepatic tissue samples weighing 100 mg were homogenized in 2.5 mL of ice-cold double-distilled water. After determining the total protein concentration in homogenates, 2 mL of homogenates were hydrolyzed with HCl (final concentration: 6 M) at 105°C for 18 h. Hydrolysates were filtrated with 3 mm filter paper and dried at 40°C. The samples were then incubated with Ehrlich's solution (25% (w/v) p-dimethylaminobenzaldehyde and 27.3% (v/v) perchloric acid in isopropanol) at 50°C for 90 min and measured at A558 nM. All results were normalized by total protein concentration and calculated using a standard curve.

### 2.6. Tissue Specimen and Sample Preparation for 2DE

Three liver samples were selected from each group and homogenized in liquid nitrogen-cooled mortar and pestle and then dissolved in lysis buffer (8 M urea, 4% CHAPS, 40 mM Tris, 65 mM DTT). Samples were sonicated on ice for 10 sec, three times in an ultrasonic processor and centrifuged for 1 h at 20,627 ×g (15,000 RPM) to remove DNA, RNA, and any particulates. The concentrations of all samples were measured by a modified Bradford assay (Bio-Rad). The extracts from the same group were pooled with equal amounts and the concentrations were measured again. All samples were stored at −80°C until further processed.

### 2.7. Two-Dimensional Electrophoresis (2DE) and Image Analysis

2DE and image analysis was performed according to previously described methods [18] with some modifications. Briefly, the first-dimensional isoelectric focusing (IEF) step was accomplished on an IPGphor IEF system (Amersham Biosciences, Uppsala, Sweden). 100 *μ*g of total proteins for analytical or 1.0 mg for preparative runs were mixed with a rehydration solution (8 M Urea, 2% CHAPS, 18 mM DTT, 0.5% IPG buffer, and bromophenol blue) and applied to Immobiline pH-gradient IPG dry strips (IPG buffer, pH 3–10). After rehydration for 12 h in 250 *μ*L of rehydration buffer containing the protein samples, proteins were focused successively for 1 h at 500 V, 1 h at 1000 V, and 10 h at 8000 V on an IPGphor. After IEF, IPG strip was equilibrated for 2 × 15 min in 50 mM Tris-HCl, pH 8.0, 6 M urea, 30% glycerol, 2% SDS, and bromophenol blue containing buffer. DTT (1%) was added to the first equilibration buffer. In the second equilibration buffer, DTT was replaced by 2.5% iodoacetamide (IAA), and the second dimension separation was performed with 12% sodium dodecylsulfate-polyacrylamide gel electrophoresis (SDS-PAGE) in Ettan DALT II electrophoresis apparatus. The analytical gels were visualized with silver staining, while the preparative gels were stained with Coomassie Blue G250 (Bio-Rad). The silver-stained 2-D gels were scanned at an optical resolution of 84.7 um/pixel using a GS-710 imaging densitometer (Bio-Rad). Spot detection, quantification, and matching were performed using ImageMaster software (GE healthcare, USA). Quantitative analysis was performed using the Student's *t*-test between normal and model groups or model and FZHY groups with a level of 95%.

### 2.8. In-Gel Digestion

For MS fingerprinting, gel plugs were cut out off the preparative Coomassie blue-stained gels, destained with 100 mM NH_4_HCO_3_ in 30% acetonitrile ACN, and lyophilized (VirTis Vacuum-Spin, NY, USA). The dried gel plugs were rehydrated with a total of 25 *μ*L of sequencing grade, modified trypsin (Promega, Madison, USA) in 100 mM ammonium bicarbonate at 47°C for 2 h. Then 20 *μ*L of 50 mmol/L NH_4_HCO_3_, pH 8.3 was added, and the gel slices were incubated at 37°C for 12 h. The digestion buffer was removed and saved. The gel pieces were extracted with 200 *μ*L of 60% ACN/0.1% TFA for 15 min with sonication, and the supernatant was removed. The extraction was repeated twice more and the three extracts plus the first saved digestion buffer fraction were pooled and dried completely under vacuum. This in-gel digestion method was mainly performed according to the method described previously [19] with the modifications as described previously.

### 2.9. MALDI-TOF MS Identification and Database Search

Peptide mixtures of each gel plug were redissolved in 0.1% TFA, desalted, and concentrated by ZipTips (Millipore, Boston USA). Peptide solution (0.75 mL) was mixed with 0.75 mL of matrix (CHCA in 30% ACN/0.1% TFA), spotted on a target disk, and allowed to air-dry. Samples were analyzed using a Bruker Reflex III MALDI-TOF mass spectrometer (Karlsruhe, Germany). Protein database search was performed by the MASCOT search engine (http://www.matrixscience.com/; Matrix Science, London, UK) using monoisotopic peaks against the NCBI nonredundant protein database (http://www.ncbi.nlm.nih.gov/) for Rattus norvegicus. Mass tolerance was allowed within 0.05%. Proteins matching more than four peptides and with a MASCOT score higher than 63 were considered significant (*P* < 0.05). 

### 2.10. Western Blot

Proteins from tissues of the normal, model, or FZHY-treated groups were subject to 12% SDS-PAGE gel electrophoresis and transferred to Hybond-C membrane (Amersham Biosciences) followed by antibody-based interrogation against vimentin antigen (vimentin, mouse monoclonal IgG; Millipore, Bedford, MA, USA.) and Glyceraldehyde 3-phosphate dehydrogenase (GAPDH) antigen (GAPDH, rabbit anti-GAPDH polyclonal IgG; Sofarbio Technology, HangZhou, China). The primary antibodies were detected by IRDyeTM800cw conjugated Goat Anti-mouse IgG (LI-COR, Inc., USA) and IRDyeTM680cw conjugated donkey anti-rabbit IgG (LI-COR, Inc., USA). Visualization of the immunoreactive proteins was accomplished using the Odyssey Infrared Imaging system (LI-COR, Inc., USA).

### 2.11. Real-Time PCR Validation

Total RNA was isolated from the liver tissues with Trizol reagent (Invitrogen, Carlsbad, CA, USA) according to the manufacturer's protocol. RNA quantity was determined by spectrophotometry, and its integrity was checked by agarose gel electrophoresis. First strand cDNA was synthesized by reverse transcription, 4 *μ*g of total RNA in a final reaction volume of 20 *μ*L using a first strand cDNA Synthesis Kit according to the manufacturer's protocol (My Cycler Thermal Cycler, USA). Primer oligonucleotide sequences specific for the real-time PCR are shown in Table 3, which were designed and synthesized by Sangon Biotech Inc. (Shanghai, China). PCR mixtures contained 1 *μ*L cDNA, 10 *μ*L SYBR Premix Ex Taq (2x, Takara, Dalian, China), and 0.25 *μ*M forward and reverse primers in a final volume of 20 *μ*L. Triplicates were performed with a Rcorbett 6.0 system (Rotor-Gene 3000, Australia) starting with a polymerase activation step for 10 s at 95°C, followed by 40 cycles of 5 s at 95°C, 15 s at 58°C, and 10 s at 72°C. Fluorescence data were acquired after each cycle. The absence of primer dimers and unspecific products was verified after every run by melting curve analysis (72 to 95°C) and agarose gel electrophoresis. 

### 2.12. Statistics

Date were expressed as mean ± SD. Statistical analysis was evaluated by one-way analysis of variance (ANOVA), followed by the Student-Newman-Keuls test for multiple comparisons, which was used to evaluate the difference between two groups. *P* < 0.05 was considered to be significant.

## 3. Results and Discussion

### 3.1. FZHY Protects against Hepatic Injury and Hepatic Fibrosis in DMN-Induced Rats

**Figure 1:**
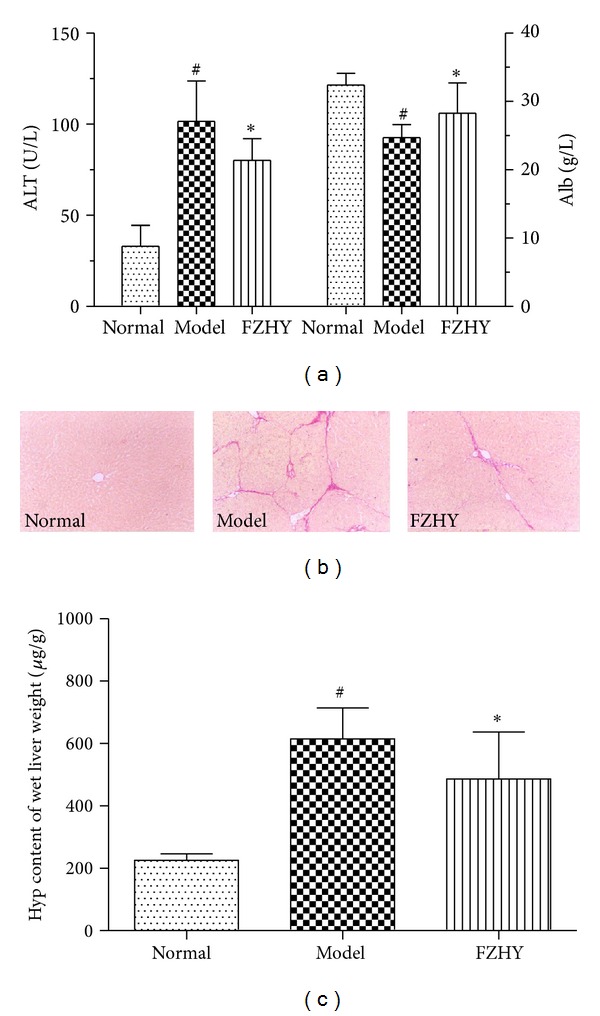
Hepatic injury and fibrosis are alleviated in FZHY-treated DMN rats. (a) Serum ALT and albumin levels were analyzed using commercial kits (*n* = 8). (b) Representative images of collagen decomposition in each group. Sirus red staining. (c) Quantitative analysis of the Hyp content in liver tissues in each group (*n* = 8). ^#^ *P* < 0.05 versus normal control mice (normal), *P < 0.05 versus DMN-induced mice (model).

Rats being injected with DMN for 4 weeks and then recovering for another 4 weeks (model rats) still developed severe hepatic injury in the liver, reflected by the elevating ALT level compared to control rats; however, rats treated with FZHY showed a lower level, the albumin in DMN-induced rats decreased compared to control mice, while FZHY improved the albumin level (Figure 1(a)). As shown in Figure 1(b) by sirus red staining, model rats developed fibrosis in the liver and FZHY administration greatly reduced accumulation of collagen in the tissue. Similarly, the Hyp content was significantly greater in the model rats' liver compared to control rats. FZHY treatment, however, remarkably decreased the Hyp content in the livers of model rats (Figure 1(c)). These findings show that FZHY exerted good effects ameliorating hepatic injuries and fibrosis in DMN-induced rats, so next we were eager to know how FZHY did that.

### 3.2. Differential 2DE Analysis of Liver Tissue Proteins

**Figure 2:**
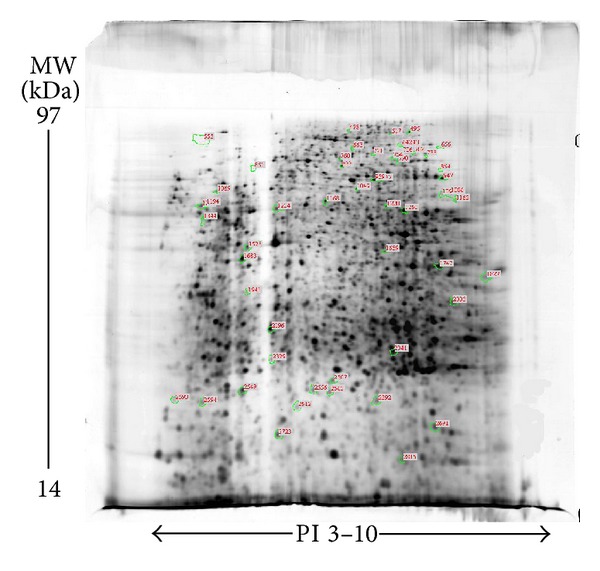
Differential proteins spots of rat liver proteins (model group) on 2DE gel. First dimensional separation was performed on an immobilized nonlinear pH 3–10 strip, followed by the second-dimensional separation on 12% SDS-PAGE. The protein spots were visualized by silver staining. A total of 54 protein spots showed significantly different expression between normal and model groups by image analysis and comparison and are marked with green circles and red ID codes.

**Figure 3:**
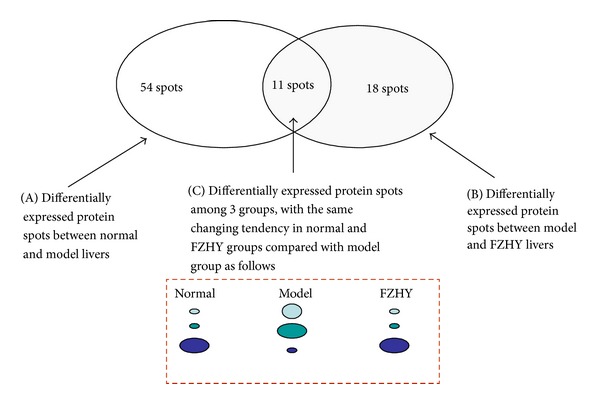
Grouping of differentially expressed protein spots. Through comparison of 2DE gels between two groups with a gel-in model as a reference, there were 54 differential spots between normal and model groups (pattern A), 18 differential between model and FZHY groups, and 11 spots were overlapped between pattern A and pattern B, in which normal and FZHY groups had the same tendency of differential expression compared with ones in model group (pattern C).

**Figure 4:**
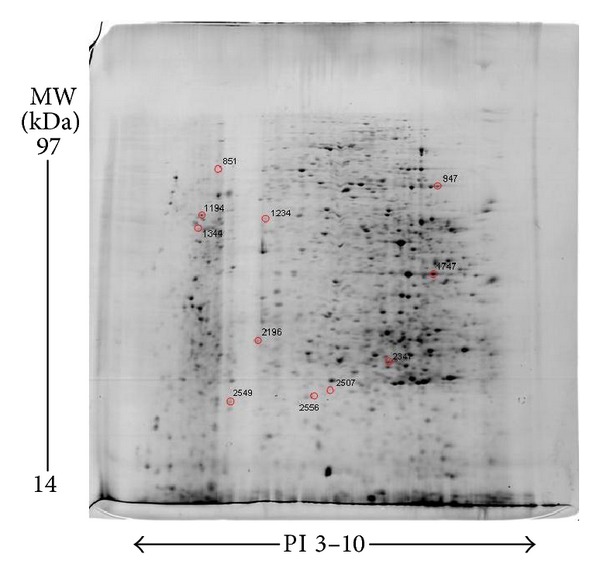
The differentially expressed 11 spots among all 3 groups on 2DE gel from a preparative gel. The 11 spots marked with red circles and ID codes expressed among all three groups. More importantly, the 11 spots in the normal and FZHY group showed the same differential expression compared with the model group. 1.0 mg of total protein was isoelectrically focused on IPG strips (pH 3–10), then separated by 12% SDS-PAGE as seconddimension. The protein spots were visualized by Coomassie Blue G250 staining.

The 2DE gel shows a typical separation of liver tissue proteins (total) in normal, model, and FZHY groups into 1000~1200 spots. The 2DE experiment was repeated three times. Nine 2DE gel images were analyzed, and one of the most reproducible images from model group tissue sample was selected as a reference gel. With ImageMaster software, ratios of normalized spot intensities of normal to model control tissue or model to FZHY tissue were calculated. A total of 61 protein spots exhibited significant intensity changes as the gels between normal and model or between model and FZHY were image analyzed and compared (*P* < 0.05) (Figure 2). We grouped these 61 differentially expressed protein spots into three major patterns (Figure 3): pattern A: 54 spots differentially expressed between normal and model group; pattern B: 18 spots differentially expressed between model and FZHY group; pattern C (Figure 4): 11 spots were overlapped between pattern A and pattern B, which means these 11 spots were differentially expressed among all 3 groups. More importantly, the 11 spots in the normal and FZHY groups showed the same differential expression compared with the model group. For example, when a spot intensity in the normal group was decreased compared to model, the corresponding spot intensity in FZHY also decreased.

### 3.3. Identification of the Differentially Expressed Proteins by MS

Among the previously identified 61 differentially expressed protein spots, the 23 spots showing the largest difference were excised from the preparative gels, followed by in-gel tryptic digestion. 21 peptide mass fingerprints (PMFs) were successfully identified through analysis with MALDI-TOF MS and through protein database searching; 15 PMFs matched the database information and 6 PMFs failed. Interesting to note that three different spots (spots no. 1194, 1334, 1208) were identified as vimentin, among them spots 1069 and spot 1194 were identified as vimentin isoforms. Therefore we actually obtained a total of 14 differentially expressed proteins, among them, 12 belonged to pattern A, 9 belonged to pattern B, while 8 proteins were overlapped between patterns A and B and belonged to pattern C. The protein IDs and descriptions are shown in Tables 4 and 5. 

### 3.4. The Differentially Expressed Proteins between Normal and Fibrotic Liver

Liver fibrosis is orchestra of multiplex disorders involved in many liver cells and cytokines [20]. Although recent years have witnessed big progresses in understanding the mechanism of liver fibrosis, including elucidating the pivotal role of hepatic stellate cell activation and transforming growth factor-*β*1 in the formation of liver fibrosis, the complicated mechanism of fibrogenesis is not yet fully understood. The proteomics analysis of liver fibrotic animals and cells could provide useful information for understanding liver fibrogenesis, finding potential diagnostic markers and discovering therapeutic target candidates [21–26]. In the present study, we used a 2DE-based proteomic approach to separate liver proteins and MALDI-TOF MS for their identification in distinct proteomes of normal rat liver and fibrotic liver. Our result showed that 12 proteins were expressed differentially between normal and fibrotic (model) livers as listed in Table 4, which were mainly involved in five biological aspects, such as substance metabolism, protein binding, oxidative stress, stress response and cellular calcium ion homeostasis. Up- and downregulated proteins were classified by the biological processes in which they were supposed to be involved according to gene ontology criteria (http://www.ebi.ac.uk/Databases/ontology.html). Compared to the normal liver, the fibrotic liver had the decreased expression levels of catalase, clathrin light chain, regucalcin, fructose-bisphosphate aldolase B, and aldo-keto reductase family 1, but increased levels of vimentin, Glutathione S-transferase, Aldehyde dehydrogenase, *γ*-actin and S-adenosylhomocysteine hydrolase, isoform, and so forth. Among these differential expressed proteins, aldehyde dehydrogenase [27], fructose-bisphosphate aldolase B [28], vimentin [29], heat shock protein 90-*β* [21], catalase [30], and glutathione S-transferase [31] had already been described in the context of fibrogenesis; others are not reported to have link with liver fibrosis. Regucalcin was related to cellular calcium ion homeostasis, which was reportedly involved in chronic liver injury and acute liver failure [32–34] through regulating ATPase activity and calcium-mediated signaling, which may be involved in fibrosis. Other proteins, including aldo-keto reductase family 1, member D1, S-adenosylhomocysteine hydrolase, and clathrin light chain, even have not been reported to be linked to liver damage or cirrhosis. 

### 3.5. The Differentially Expressed Proteins between Fibrotic Liver and FZHY-Treated Liver and Validation by Western Blotting and Real-Time PCR

We also identified 9 differentially expressed proteins between the model and FZHY groups (Table 4). Among them, 8 proteins were also present as differentially expressed proteins between normal and model samples as shown in Table 5. More importantly, these 8 differentially expressed proteins among 3 groups as pattern C had very interesting feature; all of 8 proteins in normal and FZHY groups had the same tendency of differential expression compared with the ones in model group. For example, normal group had a very lower expression of vimentin, compared to model, however, and FZHY group had decreased vimentin level too. It indicates that FZHY could restore proteins expressions which were expressed abnormally in fibrotic liver, and these differentially expressed proteins among 3 groups provide new insights into elucidation of FZHY action mechanism against liver fibrosis.

**Figure 5:**
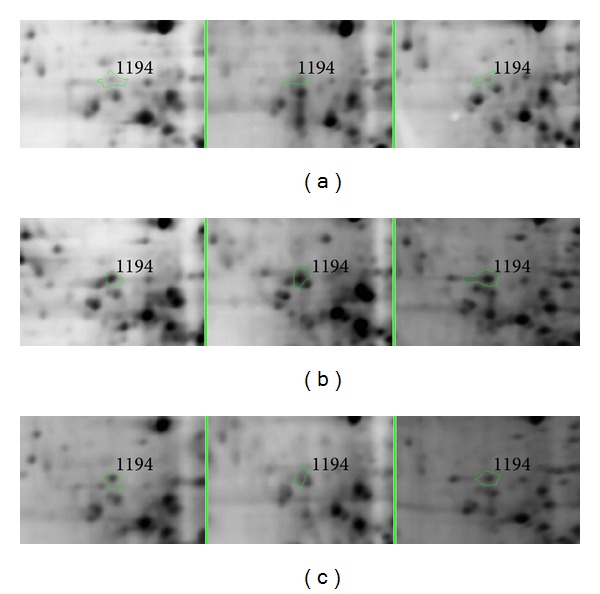
Amplification of focal 2DE to identify protein spot 1194*-*vimentin (the spot marked with a green circle). (a) Normal group; (b) model group; (c) FZHY group. The spot 1194 expression in model group was significantly increased.

**Figure 6:**
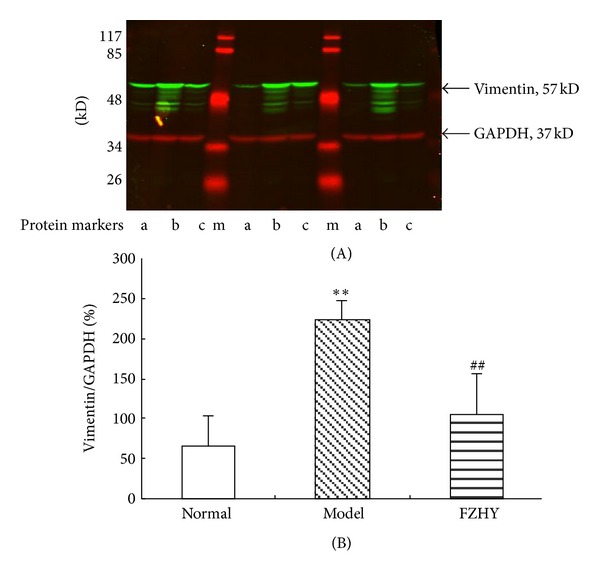
Protein expression of vimentin in liver tissues by western blot analysis. (A) Immunoblotting with a vimentin monoclonal antibody and with Glyceraldehyde 3-phosphate dehydrogenase (GAPDH) as internal reference, following SDS-PAGE. (a), normal group; (b), model group; (c), FZHY group; (m), protein molecular marker. (B) Graphic representation of the relative expression of vimentin. The values are represented as the density of vimentin versus GAPDH (%). **P < 0.01  *versus *normal; ^##^ *P* < 0.01  *versus *model spots.

To confirm the previous presented results, vimentin, one of differentially expressed proteins was selected to be validated by western blotting and PCR.  Figure 5 showed vimentin with higher expression in the model group but significantly downregulated in the normal and FZHY groups in 2DE gels. As shown in Figure 6, the western blot results of vimentin were consistent with results of the 2DE gels. To examine whether protein alterations observed by proteomic analysis correlate with the changes of the respective mRNAs at the transcription level, vimentin was also chosen for further validation by real-time PCR. As shown in Table 6, the expression of vimentin mRNA dramatically increased in model group (*P* < 0.01), while significantly decreased in FZHY group (*P* < 0.05). 

Vimentin, as the major intermediate filament protein with function of skeleton organization, is not only a kind of matrix component which contributes to fibrosis formation, but also a marker of mesenchymal cells [35, 36]. The effector cell for liver fibrogenesis is myofibroblast, which can come from hepatic stellate cell (HSC) activation and epithelial cells such as hepatocyte transformation through the process of epithelial-to-mesenchymal transition named as EMT [37]. HSC activation and epithelial cells EMT both increased the expression of vimentin dramatically [36, 38]. Therefore, in the study, FZHY inhibits the increase vimentin and its isoform expression, and it not only reconfirms the FZHY efficacy on liver fibrosis [16], but also suggests that FZHY action mechanism is related to inhibiting HSC activation or EMT in liver cells. Among other 7 proteins regulated by FZHY, gamma-actin had similar function as vimentin. Aldehyde dehydrogenase 1 family member A1, fructose-bisphosphate aldolase B, aldo-keto reductase family 1 member D1, S-adenosylhomocysteine hydrolase isoform, and HSP90 are related to stress response and substance metabolism including retinoic acid, carbohydrate, and bile acid. In the study, FZHY could influence the oxidative stress in liver, which is consistent with our previous report [39]. However, it is the first time to know that FZHY can modulate the substance metabolism in fibrotic liver and cells, which maybe is a new action mechanism of FZHY against liver fibrosis. Chaperonin subunit 8 was only differentially expressed between model and FZHY groups, and the validation and significance would need further exploration. 

Although two-dimensional gel provides high-resolution separation, it has a number of shortcomings. It is difficult to identify proteins of certain types, in particular, proteins with low abundances, membrane protein, and proteins at extreme of molecular size, while mammalian tissue has complex and high dynamic abundance ranges of proteins, which increase the challenge for the effective detection of low-abundance proteins such as transcription factors and cytokines. Although we get some new and valuable insights in the mechanism of liver fibrosis and FZHY antifibrotic action in the study, we failed to characterize those well-identified proteins (e.g., TGF-*β*, MMPs, and TIMPs) closely involved in fibrogenesis. This remains a common theme among studies of the liver proteome and emphasizes the importance of implementing additional strategies (e.g., subcellular fractionation, glycoproteome, or cysteinyl subproteome enrichment) to reduce sample complexity, improve proteome coverage, and enhance the detection of low-abundance proteins important to the study of mechanism of liver fibrosis and therapeutic drug target [40]. And of course we still need combine the conventional approaches such as western blot for investigating pharmacological mechanisms.

## 4. Concluding Remarks

The current study demonstrated that there are 12 proteins responsible for fibrogenesis induced by DMN in rats; the roles of regucalcin, aldo-keto reductase family 1, member D1, S-adenosylhomocysteine hydrolase, clathryn light chain in the liver fibrogenesis were not clear yet. Also among them, 8 proteins in fibrotic liver were regulated by FZHY, including aldehyde dehydrogenase, vimentin isoform (CRA_b), gamma-actin, vimentin, fructose-bisphosphate aldolase B, aldo-keto reductase, S-adenosylhomocysteine hydrolase isoform, and HSP90. It indicates that the action mechanism of FZHY antiliver fibrosis may be associated with modulation of proteins associated with metabolism and stress response, as well as myofibroblast activation. The study provides new insights and data for exploring the liver fibrogenesis pathophysiology and FZHY action mechanism against liver fibrosis.

## Figures and Tables

**Table 1:** Composition of Fuzheng Huayu recipe (FZHY).

Herbal components	g
*Radix Salviae Miltiorrhizae *	8.0
*Fermentation Mycelium Powder *	4.0
*Fructus Schisandrae Chinensis *	2.0
*Semen Persicae *	2.0
*Pollen Pini *	2.0
*Gynostemma Pentaphyllammak *	6.0

Amount in 24 g of Fuzheng Huayu recipe.

**Table 2:** Quality control standard for Fuzheng Huayu recipe (FZHY).

Compounds (marker)	Quality criterion
Salvianolic acid B	Referred to *Radix Salvia Miltiorrhizae*, should not be less than 3.15 mg in 1 g of extract of FZHY recipe powder
Sodium Danshensu	Referred to *Radix Salvia Miltiorrhizae*, should not be less than 2.75 mg in 1 g of extract of FZHY recipe powder
Adenosine	Referred to *Mycelium powder*, should not be less than 1 mg in 1 g of extract of FZHY recipe powder
Schisandrin B	Referred to *Fructus Schisandrae Chinensis*, should not be less than 0.475 mg in 1 g of extract of FZHY recipe powder

**Table 3:** Primers used for real-time PCR.

Gene	Primer sequences (5′-3′)	Gen bank accession number	Length (bp)
Vimentin	Sense: CTT CGA AGC CAT GTC CAC CA	NM-031140	200
	Antisense: 5′-CAC CGA ACA TCC TGC GGT AG		
*β*-actin	Sense: TGA CGA GGC CCA GAG CAA GA	DQ237887	331
	Antisense: ATG GGC ACA GTG TGG GTG AC		

**Table 4:** Identification of 15 differentially expressed proteins among normal, model, and FZHY groups.

Spot number	NCBI GI	Molecular weight	PI	Sequence coverage %	Protein description
733^A^	6978607	60062	7.07	21	Catalase ↓
967^A^	203361	23220	4.63	26	Clathryn light chain ↓
1069^A^	149021114	53725	5.06	31	Vimentin isoform CRA_b ↑
1683^A^	408807	33938	5.40	38	Regucalcin ↓
2563^A^	58331159	25388	8.42	18	Glutathione S-transferase Yc2 subunit ↑
947^AB^	14192935	54994	7.94	29	Aldehyde dehydrogenase 1 family, member A1; ↑
1194^AB^	149021114	53725	5.06	30	Vimentin isoform CRA_b ↑
1234^AB^	109492380	59163	5.67	22	Gamma-actin ↑
1334^AB^	14389299	53757	5.06	21	Vimentin ↑
1747^AB^	1619606	40035	8.66	24	Fructose-bisphosphate aldolase B ↓
2341^AB^	20302063	37639	6.18	20	Aldo-keto reductase family 1, member D1 ↓
2549^AB^	149030911	44800	6.08	14	S-adenosylhomocysteine hydrolase, isoform ↑
2556^AB^	256089	83606	5.06	10	HSP90 ↑
706^B^	149059759	60121	5.38	15	Chaperonin subunit 8(theta) ↓
1208^B^	149021114	53725	5.06	24	Vimentin isoform CRA_b ↓

^A^means Pattern A, in which the spot proteins differentially expressed between normal and model groups.^B^means Pattern B, in which the spot proteins differentially between FZHY and model groups. The upward arrow (↑) or downward arrow (↓) indicates the spot proteins in the model group had increased or decreased expression compared to ones in normal or FZHY group, respectively.

**Table 5:** Differentially expressed proteins among 3 groups (pattern C).

Spot number	Protein	Ratio	
		Normal versus model	FZHY versus model
947	Aldehyde dehydrogenase 1 family, member A1	1.48 ↓	1.02 ↓
1194	Vimentin isoform CRA_b	1.93 ↓	1.82 ↓
1234	Gamma-actin	1.14 ↓	1.22 ↓
1334	Vimentin	100000 ↓	100000 ↓
1747	Fructose-bisphosphate aldolase B	1.80 ↑	1.01 ↑
2341	Aldo-keto reductase family 1, member D1	1.92 ↑	1.27 ↑
2549	S-adenosylhomocysteine hydrolase, isoform	1.70 ↓	1.07 ↓
2556	HSP90	2.19 ↓	1.17 ↓

(1) Pattern C indicates the proteins differentially expressed among all 3 groups; moreover, those proteins in normal and FZHY groups had the same tendency of differential expression compared to the ones in model group. (2) Ratio value was calculated by the 2-DE analysis software while the spots in model group as a reference. The upward (↑) indicates the increased and downward (↓) indicates the decreased spot expression level compared to the ones in model group.

**Table 6:** Semiquantitative expression of vimentin mRNA in liver tissues by real-time PCR ($\bar{x}\pm s$).

Group	*n*	Vimentin
Normal	3	0.99 ± 0.07
Model	3	1.87 ± 0.25**
FZHY	3	1.13 ± 0.16^#^

**P < 0.01, versus normal group. ^*#*^ *P* < 0.05, versus model group.

## Abbreviations

2DE: Two-dimensional gel electrophoresis
DMN: Dimethylnitrosamine
DTT: Dithiothreitol
ECM: Extracellular matrix
FZHY: Fuzheng Huayu recipe
GAPDH: Glyceraldehyde 3-phosphate dehydrogenase
HSC: Hepatic stellate cell
IPG: Immobilized PH gradient
IEF: Isoelectric focusing
MALDI-TOF MS: Matrix-assisted laser desorption ionization time of flight mass spectrometry
PAGE: Polyacrylamide gel electrophoresis
PMF: Peptide mass fingerprint
PCR: Polymerase chain reaction
SDS: Sodium dodecyl sulfate
TCM: traditional Chinese medicine.
